# Genome-wide identification of sugar transporter gene family in Brassicaceae crops and an expression analysis in the radish

**DOI:** 10.1186/s12870-022-03629-2

**Published:** 2022-05-18

**Authors:** Tongjin Liu, Chonglai Bao, Qiuyan Ban, Changyi Wang, Tianhua Hu, Jinglei Wang

**Affiliations:** 1 College of Horticulture, Jinling Institute of Technology, Nanjing, 210038 China; 2 Institute of Vegetable, Zhejiang Academy of Agricultural Sciences, Hangzhou, 310021 China

**Keywords:** Sugar transporter proteins (STPs), Gene family, Radish, Expression analysis, Biotic and abiotic stress

## Abstract

**Background:**

Sugar not only is an important biomacromolecule that plays important roles in plant growth, development, and biotic and abiotic stress tolerance but also provides a skeleton for other macromolecules, such as proteins and nucleic acids. Sugar transporter proteins (STPs) play essential roles in plant sugar transport and ultimately affect the abovementioned life processes. However, the evolutionary dynamics of this important gene family in Brassicaceae crops are still largely unknown, and the functional differentiation of radish *STP* genes remains unclear.

**Results:**

In the present study, a comparative genomic study of *STP* genes in five representative Brassicaceae crops was conducted, and a total of 25, 25, 28, 36 and 49 *STP* genes were individually identified in *Raphanus sativus* (Rs), *Brassica oleracea* (Bo)*, B. rapa* (Br)*, B. napus* (Bn) and *B. juncea* (Bj), which were divided into four clades by phylogenetic analysis*.* The number of *STP* genes was no direct correlation with genome size and the total number of coding genes in Brassicaceae crops, and their physical and chemical properties showed no significant difference. Expression analysis showed that radish *STP* genes play vital roles not only in flower and seedpod development but also under heavy metal (cadmium, chromium and lead), NaCl and PEG-6000 stresses, *Agrobacterium tumefaciens* infection, and exogenous sugar treatment. *RsSTP13.2* was significantly upregulated in the resistant radish cultivar by *A. tumefaciens* infection and induced by heavy metal, NaCl and PEG-6000 stress, indicating that it is involved in resistance to both biotic and abiotic stress in radish.

**Conclusions:**

The present study provides insights into the evolutionary patterns of the STP gene family in Brassicaceae genomes and provides a theoretical basis for future functional analysis of *STP* genes in Brassicaceae crops.

**Supplementary Information:**

The online version contains supplementary material available at 10.1186/s12870-022-03629-2.

## Background

Most plants on earth, except for parasitic plants, fix carbon through photosynthesis to produce soluble sugar as the main carbohydrate through various reactions in the cytoplasm [1]. These sugars have the functions of providing energy for cell life activities, providing a skeleton for macromolecules such as proteins and nucleic acids. Sugars also participate in the regulation of various metabolic pathways and biotic and abiotic stress responses of plants as signal molecules [2, 3].

Sugars synthesized from source cells were transported to the sink organs via the phloem mainly in the form of sucrose [4]. In the sink organs, sucrose can directly enter the cells by symplast pathways through plasmodesmata or via the apoplast pathway mediated by sugar transporters [1, 4]. In addition, sucrose can also be hydrolysed to monosaccharides glucose and fructose in the apoplast by cell wall invertases, and thereafter, was transported into the cells across the plasma membrane mediated by monosaccharide transporters [4]. At present, three types of sugar transporters have been found in plants: sugars will eventually be exported transporters (SWEETs), monosaccharide transporters (MSTs) and sucrose transporters (SUTs) [5]. MSTs can be further divided into seven subfamilies, including sugar transporter protein (STP), vacuolar glucose transporter (VGT), tonoplast monosaccharide transporter (TMT), plastidic glucose transporter (GlcT)/suppressor of G protein beta 1 (SGB1), polyol transporter (PLT), inositol transporter (INT) and early response to dehydration-6-like (ERD6L) [6]. Among these subfamilies, the STPs are the best characterized subfamily and function in transporting hexose from the apoplastic space into the cell [6].

STPs contain 12 transmembrane domains and are located on plasma membranes, regarded as H^+^/sugar symporters [7]. There are 14 members (*AtSTP1*-*AtSTP14*) of the STP gene family in the *Arabidopsis* genome [8], and *AtSTP1* is the first sugar transporter cloned from a higher plant [9]. To date, all 14 AtSTPs have been studied by heterologous expression in yeast, and most of them have a broad spectrum of substrates. AtSTP1 [9], AtSTP2 [10], AtSTP4 [11], AtSTP6 [12], AtSTP11 [13] and AtSTP13 [4, 14] were proven to transporter hexose glucose, galactose and mannose, pentose xylose and nonmetabolisable 3-O-methylglucose, while AtSTP9 is a glucose-specific transporter [15]. AtSTP7 cannot transport the abovementioned monosaccharides but is specific for the pentoses l-arabinose and d-xylose [6, 16], and AtSTP3 [8] has uptake activity for the abovementioned monosaccharides except for fructose. AtSTP5 might be a pseudogne [17]. *AtSTP10* encodes a protein that catalyses the uptake of glucose, galactose and mannose but not fructose [18]. AtSTP8 was reported to have broad-spectrum monosaccharides such as glucose, fructose, mannose, galactose, arabinose and xylose [16, 17]. AtSTP12 can transport glucose, galactose and mannose [16], while AtSTP14 does not take up glucose and fructose but can transport galactose [19].

Previous studies have indicated that STPs are involved in regulating multiple growth and developmental processes and biotic and abiotic resistance by regulating the distribution and accumulation of soluble sugars in plants. Overexpression of *AtSTP1* in *A. thaliana* significantly alters the extracellular sugar contents and inhibits its growth and branching [20]. *AtSTP1* and *AtSTP4* cooperate to import glucose to guard cells, providing carbon sources for light-induced stomatal opening and guarding cell starch accumulation [21]. *AtSTP4/6/8/9/10/11* displays high expression levels in pollen tubes, and a sextuple knockout plant eliminates the inhibitory effect of glucose on pollen tube elongation in vitro [22]. The expression of *AtSTP13* was significantly induced by *Botrytis cinerea* infection, and its overexpression enhanced the resistance to grey mould disease by improving glucose uptake [23], while its orthologous gene in wheat, *TaSTP13*, contributes to susceptibility to *Puccinia striiformis* f. sp. *tritici* (*Pst*), most likely by increasing the fungal sugar supply [24]. In addition, *TaSTP6* was induced in leaves by *Pst* infection, and its expression promoted the susceptibility of wheat to stripe rust [25]. A more recent study indicated that overexpression of *AtSTP8* in *A. thaliana* significantly induced the accumulation of hexose in the mature leaf and enhanced susceptibility to powdery mildew disease [17]. The expression of *STPs* is also significantly affected by abiotic stress. For example, both excess zinc and iron/zinc deficient stress significantly upregulate the expression of *STP13* in shoot of *Phaseolus vulgaris* L., while excess zinc stress upregulated and iron/zinc deficient stress downregulated its expression in roots [26]. The expression of *STP2*, *STP3*, *STP4*, *STP11*, *STP19* and *STP25* in rice was significantly induced under salt, osmotic, and drought stress. *STP11* was upregulated under ABA, IAA, 6-BA, SA and GA treatment, while *STP1* and *STP14* were upregulated under sucrose, glucose and fructose treatment [27]. These findings indicate that STPs play important roles in plant sugar transport, growth, development, and stress tolerance.

Brassicaceae includes many economically and nutritionally important crops, such as *Raphanus sativus*, *Brassica oleracea*, *B. rapa*, *B. napus* and *B. juncea.* The ancestor of diploid *Brassica* and *Raphanus* species, including *B. oleracea, B. rapa,* and *R. sativus,* has undergone Brassiceae lineage-specific whole-genome triplication (WGT) after its divergence from the *A. thaliana* lineage approximately 20 million years ago (MYA) [28]. However, the neotetraploids *B. napus* and *B. juncea* were allopolyploid between the ancestors of *B. rapa* (genome AA) and *B. oleracea* (genome CC), *B. rapa* (genome AA) and *Brassica nigra* (genome BB) [28]. The expansion and contraction of the STP gene family in these plants during their evolution are still largely unknown, even though high-quality genomes have been assembled. Thus, a genome-wide identification and analysis of the STP gene family in *R. sativus*, *B. oleracea*, *B. rapa*, *B. napus* and *B. juncea* was conducted in the present study. Additionally, the expression patterns in various organs and in response to biotic and abiotic stress were determined in the radish to characterize the functional differentiation of the STP gene family. The results of the present study provide insight into the expansion and contraction of the STP gene family in Brassicaceae and provide the basis for their functional study.

## Results

### Genome-wide identification of *STP* genes from five Brassicaceae crops

Twenty-five, 25, 28, 36 and 49 STP-encoding genes were identified from the genomes of *R. sativus* (Rs), *B. oleracea* (Bo), *B. rapa* (Br), *B. napus* (Bn) and *B. juncea* (Bj), respectively (Table S1). There were also 14 *A. thaliana STP* genes identified in this study, which were identical to those described in a previous report [4], confirming the reliability of our results. No *STP* gene was found on the scaffolds of these six genomes, while the number of *STP* genes distributed on chromosomes (Chr) of each species varied greatly. In *R. sativus*, Chr1 and Chr5 had the highest number of *STP* genes. *B. oleracea* and *B. rapa* had the most *STP* genes on chromosomes C1 and A01, respectively. *B. napus* An-subgenomes had the most genes on A03, and Cn-subgenomes had the most genes on C05 and C07. The *B. juncea* An and Bn subgenomes had the most genes on A01 and B01, respectively (Fig. S1).

**Fig. 1:**
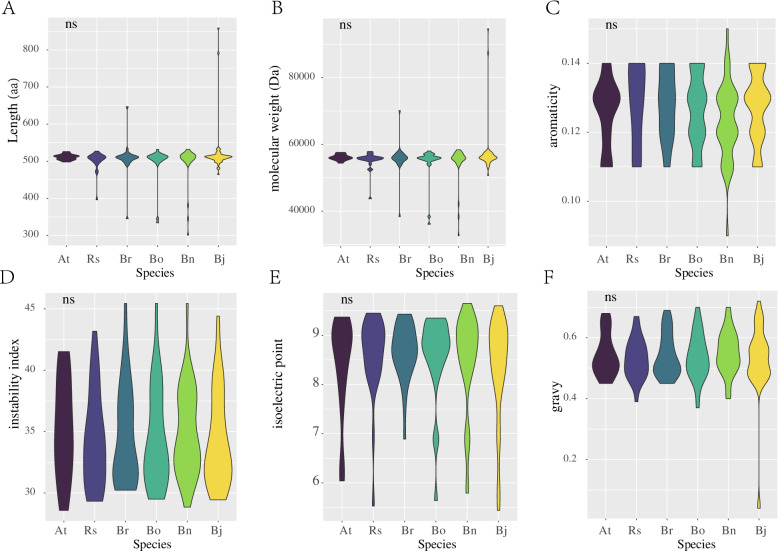
The violin diagram shows the physical and chemical properties of STP proteins in six species. (**A**) The number of amino acids of STPs. (**B**) The molecular weight of STPs. (**C**) The aromaticity of STPs. (**D**) The instability index of STPs. (**E**) The isoelectric point of STPs. (**F**) The gravy of STPs. The ‘ns’ means no significant difference

The physical and chemical properties of all STP proteins were analysed (Table S2). There were no significant differences in amino acid residue number, molecular weights, aromaticity, instability index, isoelectric point or gravy among the six species (Fig. 1). The predicted aromaticity ranged from 0.09–0.15, the instability index ranged from 28.58–45.46, and the isoelectric point ranged from 5.44–9.65 (Table S2).

### Phylogenetic relationships of STP family members from six Brassicaceae species

**Fig. 2:**
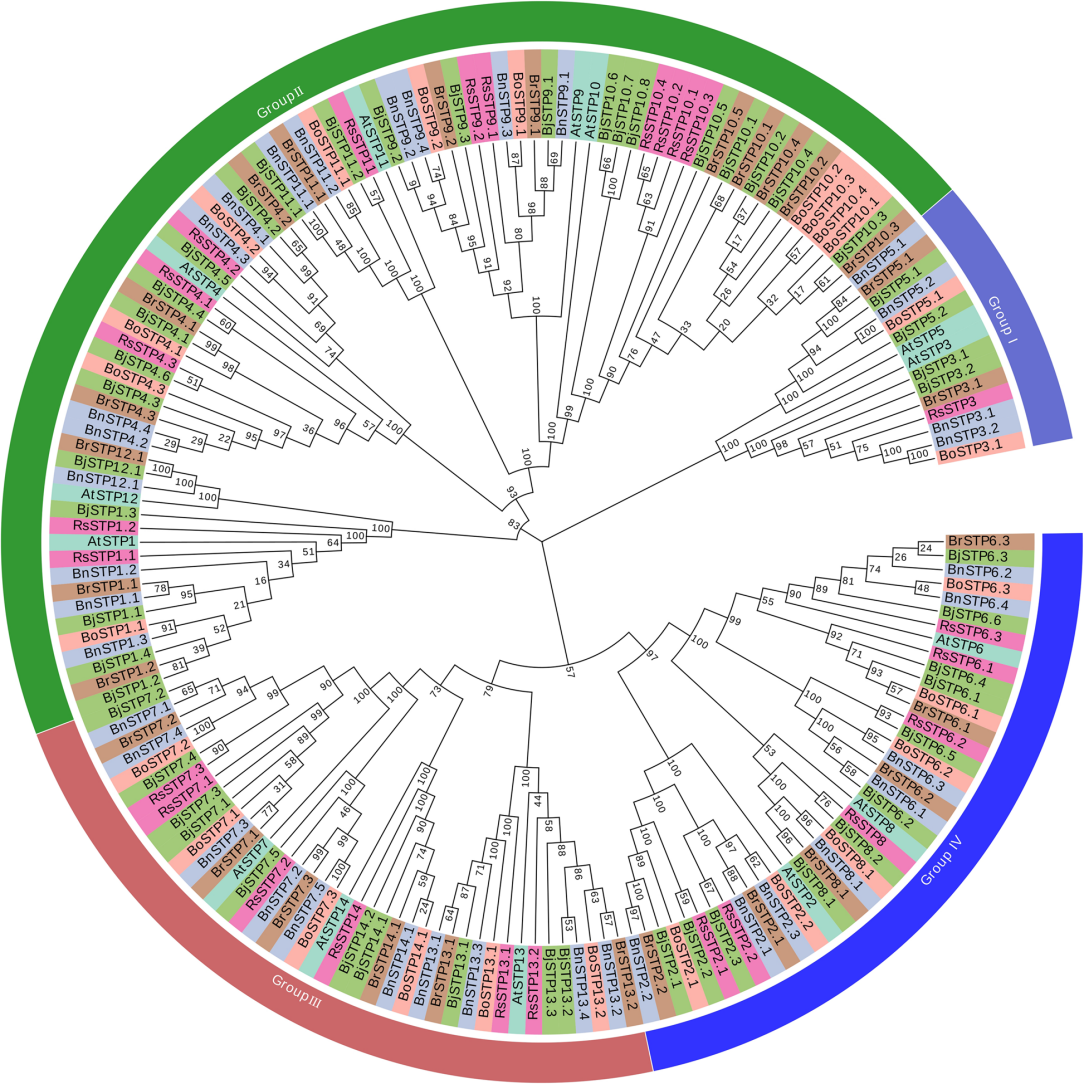
An NJ phylogenetic tree of STP protein sequences from *A. thaliana* (At), *R. sativus* (Rs), *B. oleracea* (Bo), *B. rapa* (Br), *B. napus* (Bn) and *B. juncea* (Bj). Different colours of branches represent different groups

**Fig. 3:**
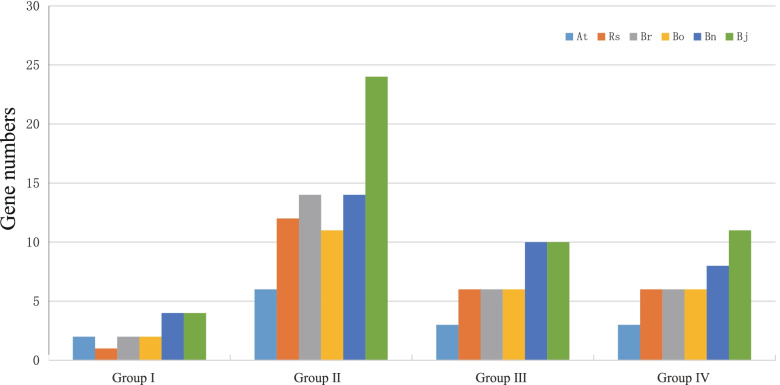
The STP gene numbers of four groups among different Brassicaceae species, including *A. thaliana* (At), *R. sativus* (Rs), *B. oleracea* (Bo), *B. rapa* (Br), *B. napus* (Bn) and *B. juncea* (Bj)

To analyse the possible evolutionary characteristics of the STP gene family in Brassicaceae, we conducted a phylogenetic tree based on 177 STP amino acid sequences from six species. All STP proteins were clustered into four groups (Fig. 2). In comparison, group II contained the most STP gene family members, followed by group III and group IV, and Group I had the fewest members (Fig. 3). For group III and IV, *R. sativus*, *B. oleracea* and *B. rapa* had the same *STP* gene number, which was between the *STP* gene number of *A. thaliana and B. napus* or *B. juncea* (Fig. 3)*.* For Group I, *R. sativus* had only one member because of the loss of *STP5*.

### Conserved motif distribution and structural analysis of the STP family

**Fig. 4:**
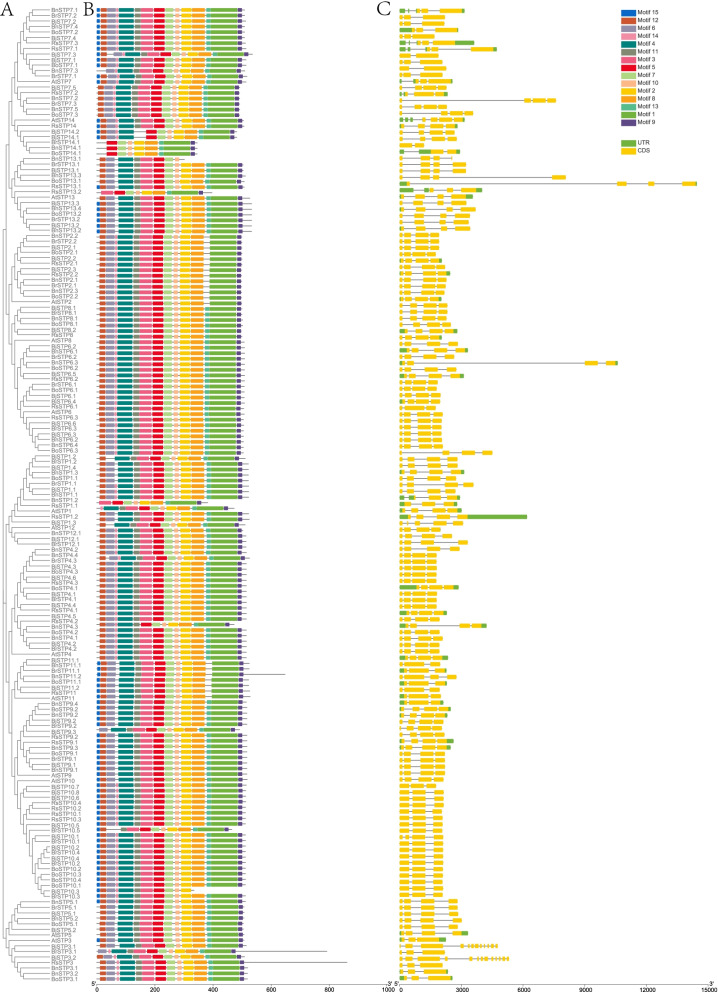
Motif and structural analyses of STPs. (**A**) Phylogenetic tree of STP proteins. (**B**) Schematic representation of the conserved motif compositions of STP. (**C**) Exon/intron structures of *STP* genes

To gain insight into potential functions and diversification among *STP*s, the encoded conserved motifs and exon–intron organizations were compared. As expected, most phylogenetically closely related STPs shared similar motifs and structures (Fig. 4). Fifteen predicted motifs were identified throughout the STP protein sequences. Motifs 1, 2, 5, 7, 8, 9, and 14 were present in all analysed STPs. The length of motifs ranged from 15 to 92, and part of the putative sugar_tr domain was predicted in motifs 1–7 and motif 11 (Table 1).

**Table 1:** Conserved motifs commonly identified in STP proteins

Motif	Protein Sequences	Length (aa)	Pfam Domain
motif1	ICIYVAGFAWSWGPLGWLVPSEIFPLEIRSAGQAINVSVNMFFTFLIAQAFLTMLCHMKFGJFFFFAGWVLVMTJFVYFLLPETKGVPIEEM	92	Sugar_tr
motif2	JPFFQQLTGINVIMFYAPVLFQTLGFGSBASLLSA	35	Sugar_tr
motif3	HENNYCKFDNQLLQLFTSSLYLAALVASFVASAVTRKYGRK	41	Sugar_tr
motif4	ANQAVPLYLSEMAPAKJRGALNIGFQLAITIGILVANLVNYG	42	Sugar_tr
motif5	KGPWGWRJSLGLAAVPALJMTJGSLFLPETPNSLJER	37	Sugar_tr
motif6	GGLJFGYDJGISGGVTSMDEFLKKFFPTVY	30	Sugar_tr
motif7	TEEAKEMLRKIRGTDBVDEEFQDJVDAS	28	Sugar_tr
motif8	TGVVNVLSTFVSIYLVDRFGRRFLFLZGGIQMLICQIIVGVIJG	44	Sugar_tr
motif9	RVWKKHWFWKRYIPD	15	
motif10	KHPWKNJLQRRYRPQ	15	
motif11	FAQNLAMLIIGRJLLGFGVGF	21	Sugar_tr
motif12	GGRAYEGKVTVFVFITCIVAA	21	
motif13	TGTLGKAYAIVVVVF	15	
motif14	KKKQAHEN	8	
motif15	MAGGAFVSEGG	11	

The exon/intron structures exhibited a highly conserved organization in *STP* genes. Most *STP*s (67%) presented four exons divided by three introns, and 28% of *STP*s had 3 exons. Three percent of *STP*s had 2 exons, and only 3 *STP*s had more than 5 exons (Fig. S2).

### Tandem duplications and synteny of *STP* genes

Segmental and tandem duplications provide critical sources of primitive genetic material for genome complexity and evolutionary novelty. We investigated the syntenic and tandem relationships of *STP* genes. In *A. thaliana*, *AtSTP4* and *AtSTP10*, distributed on chromosome 1, were demonstrated to be tandem duplications. For the other species, *STP4* and *STP10* on chromosome 1 were located in tandem duplicated regions. *STP10* had 3–5 copies in the tandem duplication cluster. However, this tandem duplication cluster was not found in *B. napus*.

**Fig. 5:**
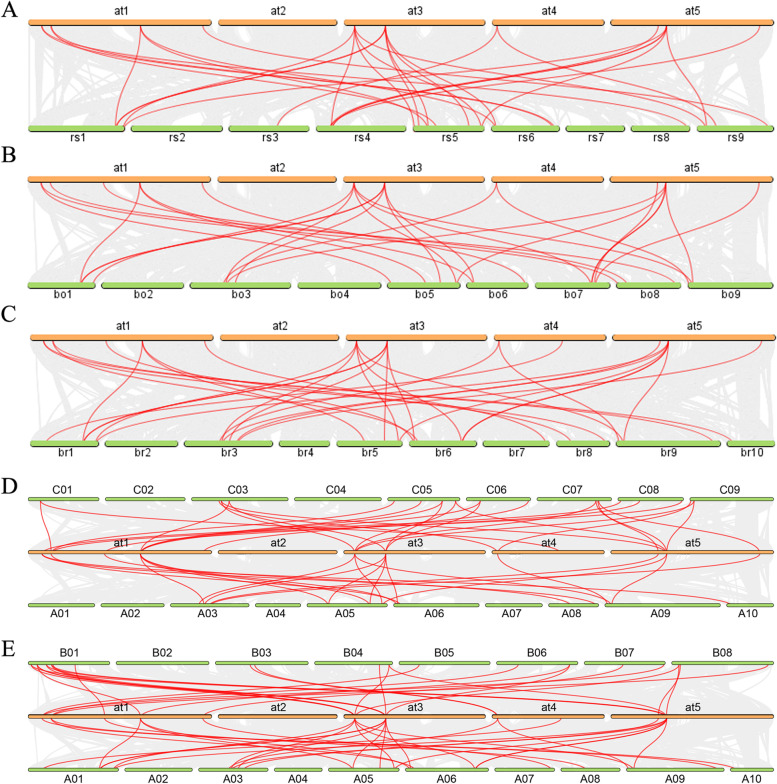
Synteny analysis of *STP* genes between *A. thaliana* and five Brassicaceae crops. The red lines highlight the syntenic *STP* gene pairs. (**A**), (**B**), (**C**), (**D**), and (**E**) indicate synteny gene pairs between *R. sativus*, *B. oleracea*, *B. rapa*, *B. napus*, *B. juncea* and *A. thaliana*, respectively

Of the 14 *AtSTPs*, most have a syntenic relationship with *STP* genes in other species, except for *AtSTP3*, which exhibited no syntenic genes in *B. juncea*, *AtSTP5*, which lacked a syntenic gene in *R. sativus*, *AtSTP10*, which had no syntenic relationship with *B. napus*, *AtSTP12,* which lacked a syntenic gene in *R. sativus* and *B. oleracea* (Fig. 5 and Table S1)*.* Additionally, most *AtSTP* genes were associated with more than one gene pair with other species. For instance, both *AtSTP4* and *AtSTP6* have more than four syntenic genes in other species. In addition, most *STP* genes in *R. sativus*, *B. oleracea*, *B. rapa*, *B. napus* and *B. juncea* have found syntenic *STP* genes in *A. thaliana* (Fig. S3)*.*

### *Cis*-acting element analysis of *RsSTPs*

To investigate the mechanism of transcriptional control of *RsSTP* genes, the *cis*-acting elements in the 1.5 kb potential promoter region of these genes were identified using the PlantCARE program. A total of 49 types of *cis*-acting elements were identified in the promoter regions of *RsSTP* genes, except for common *cis*-acting elements in promoters (e.g., CAAT boxes and TATA boxes) (Fig. S4). The *cis*-acting elements involved in light responsiveness were most abundant in the promoter regions of *RsSTPs*, indicating that they might participate in light regulatory pathways. Additionally, *cis*-acting elements responded to phytohormones such as abscisic acid (ABRE), auxin (TGA-element, AuxRE, TGA-box and AuxRR-core), methyl jasmonate (CGTCA-motif and TGAGG-motif), salicylic acid (TCA-element) and gibberellin (P-box, GARE-box and TATC-box). Furthermore, stress-related *cis*-acting elements were also found in the promoter regions, including anaerobic (ARE), drought (MBS), low-temperature (LTR), defence and stress (TC-rich repeats) and anoxic (GC motif) regions.

### Expression profiles of *RsSTPs* in different tissues

**Fig. 6:**
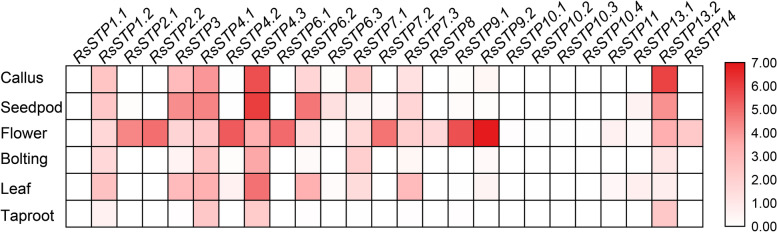
Expression profiles of radish *STP* genes in various tissues. The fragments per kilobase of transcript per million mapped reads (FPKM) data were log_2_ transformed. The color bar is shown at the right

Gene expression patterns are always associated with functional divergence in a gene family [7, 29]. Therefore, public RNA-seq data were used to analyse the expression patterns of *RsSTPs* in various tissues (taproot, leaf, bolting, flower, seedpod and callus) [30]. In the present study, the heatmap results revealed that the expression levels of nine radish *STP* genes (*RaSTP1.1/6.3/8/10.1/10.2/10.3/10.4/11/13.1*) were very low, with fragments per kilobase of transcript per million mapped reads (FPKM) < 2 in all detected tissues (Fig. 6). *RsSTP4.1* and *RsSTP4.3* were expressed in all detected tissues, while a large portion of *RsSTPs* were organ-specific. For example, *RsSTP2.1/2.2/4.2/6.1/7.2/9.1/9.2/14* were specifically expressed in flowers. The heatmap also shows the highest expression level for both *RsSTP3* and *RsSTP6.2* in seedpods and *RsSTP13.2* in calli. The diverse expression patterns suggested that *RsSTPs* might be involved in a variety of biological functions during radish growth and development.

### Expression profiles of *RsSTPs* in response to *A. tumefaciens* infection

**Fig. 7:**
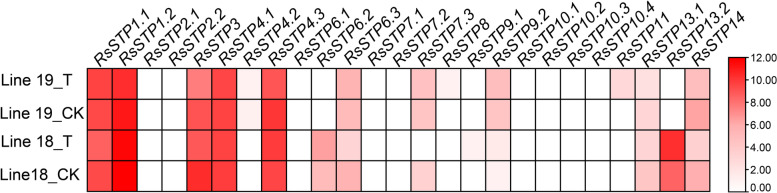
The expression profiles of *RsSTPs* 7 d after incubation with *Agrobacterium tumefaciens* in radish hypocotyls. Lines 18 and 19 are resistance and susceptible radish inbred lines to *A. tumefaciens*, respectively. CK and T represent 7 d after incubation with LB medium and *A. tumefaciens*, respectively. The fragments per kilobase of transcript per million mapped reads (FPKM) data were log_2_ transformed. The color bar is shown at the right

To clarify the response of *RsSTPs* to biotic stress, expression was compared in hypocotyls of susceptible (Lin 19) and resistant (Line 18) inbred lines inoculated with *A. tumefaciens* for 7 days by public RNA-seq data. The expression of a total of 12 *RsSTPs* (*RsSTP2.1/2.2/4.2/6.1/7.1/7.2/8/9.1/10.1/10.2/10.3/10.4*) was undetectable in both Lines 18 and 19 under the treatment and control conditions (Fig. 7). Three genes (*RsSTP1.1/1.2/4.1*) were not significantly affected, and three genes (*RsSTP3*/*13.1*/*14*) were significantly downregulated by *A. tumefaciens* infection in both lines. *RsSTP4.3* was not significantly affected in Line 18 by *A. tumefaciens* infection but was significantly downregulated in Line 19, while the expression of *RsSTP6.3* and *RsSTP7.3* was the opposite. The expression of *RsSTP6.2* and *RsSTP13.2* was significantly upregulated in Line 18 by *A. tumefaciens* infection but undetectable in Line 19. These two genes most likely confer Line 18 resistance to *A. tumefaciens* infection.

### Expression profiles of *RsSTPs* in response to cadmium (Cd), chromium (Cr) and lead (Pb) stress

**Fig. 8:**
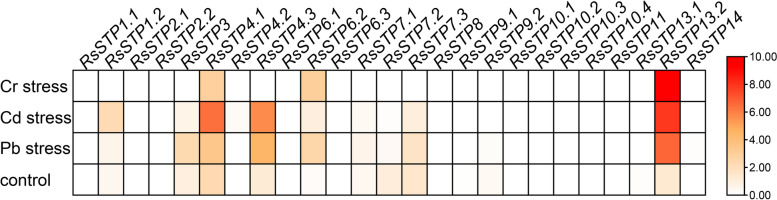
Expression profiles of *RsSTP* responses to cadmium (Cd), chromium (Cr) and lead (Pb) stress in radish. The fragments per kilobase of transcript per million mapped reads (FPKM) data were log_2_ transformed. The color bar is shown at the right

The heatmap results indicated that a large number of *RsSTPs* showed a very low expression level under the control, and most of them were not activated by heavy metal treatment, except for *RsSTP4.1*, *RsSTP4.3* and *RsSTP13.2*. For *RsSTP4.1* and *RsSTP4.3*, the expression levels were 2.5- and 15.7-fold higher under Pb stress and 21.3- and 37.4-fold higher under Cd stress than under the control, respectively, but neither was significantly affected by Cr stress. Notably, the expression levels of *RsSTP13.2* under Pb, Cd and Cr stress were 64.1-, 148.3- and 412.5-fold higher than the expression levels of *RsSTP13.2* under the control, respectively (Fig. 8). Therefore, these three significantly induced genes are most likely candidates confer radish resistance to heavy metal stress.

### Expression profiles of *RsSTPs* in response to NaCl and PEG-6000 stress and glucose, sucrose and fructose treatment

**Fig. 9:**
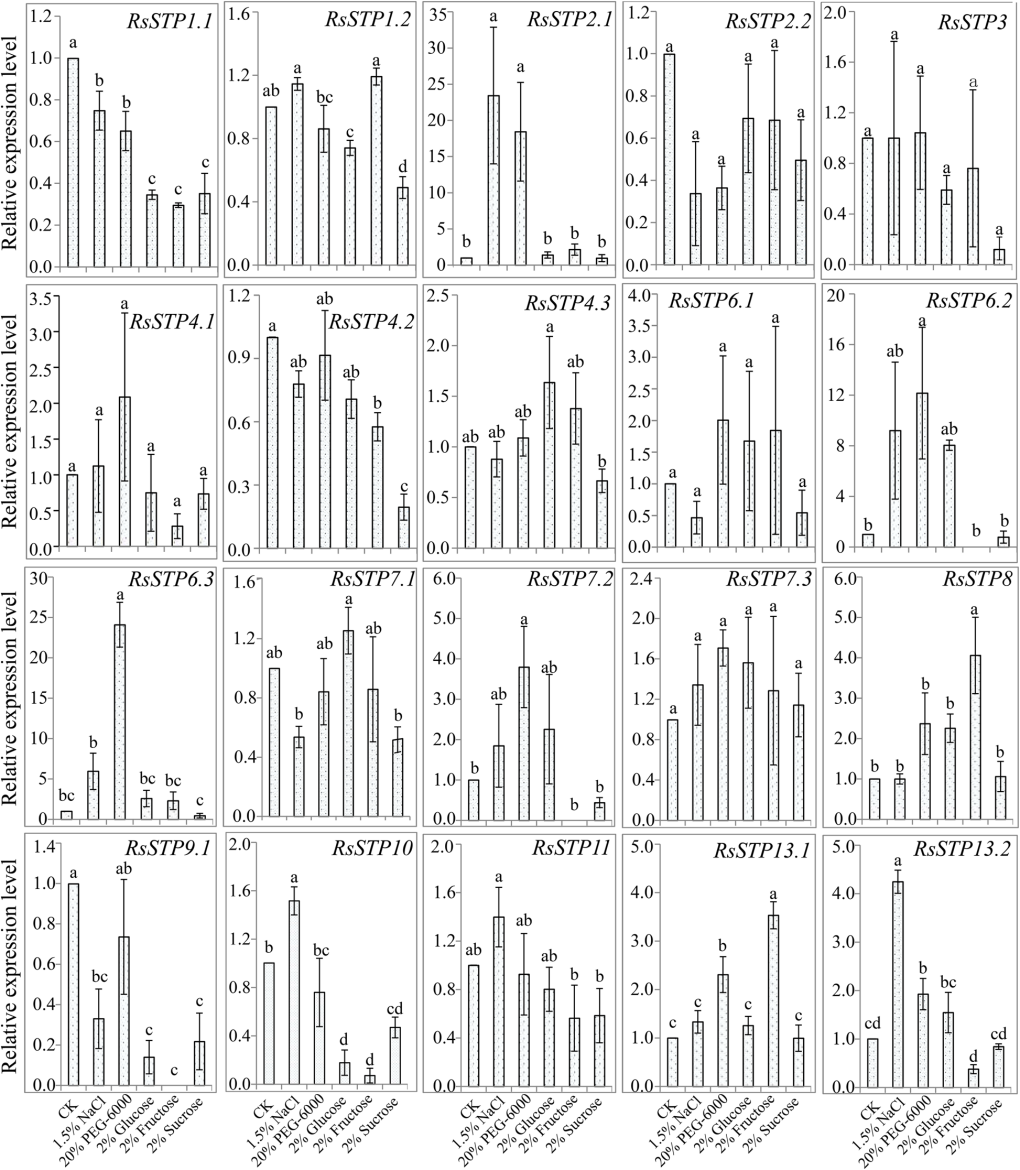
Quantitative real-time PCR analysis of *RsSTP* expression levels in the roots in response to 1.5% NaCl and 20% PEG stress and 2% glucose, 2% fructose and 2% sucrose treatment. The presented gene expression levels are relative to the expression of the reference gene *RsGAPDH*. Data are presented as the mean ± standard error of three independent experiments. CK: control treatment with distilled water

A quantitative real-time PCR (RT-qPCR) assay was performed to examine *RsSTP* expression levels in root tissue under salinity and simulated drought (PEG-6000) stress and their response to exogenous sugar treatment, including glucose, fructose and sucrose (Fig. 9). The RT-qPCR results indicated that *RsSTP9.2* and *RsSTP14* were not expressed in radish roots of either the treatment or control. The expression of eight genes, including *RsSTP2.2*, *RsSTP3*, *RsSTP4.1*, *RsSTP4.3*, *RsSTP6.1*, *RsSTP7.1*, *RsSTP7.3* and *RsSTP11*, was not significantly affected by any treatment. Three genes showed the same expression pattern in response to NaCl and PEG-6000 treatment, both of which significantly downregulated the expression of *RsSTP1.1* but upregulated the expression of *RsSTP2.1* and *RsSTP13.2*. Exogenous sugar treatment significantly affected the expression of *RsSTPs* in radish roots. All exogenous sugar treatments in the present study significantly repressed the expression of *RsSTP1.1*, *RsSTP9.1* and *RsSTP10*. In addition, the expression of *RsSTP1.2* and *RsSTP4.2* was repressed by glucose and fructose, respectively. The expression of *RsSTP8* and *RsSTP13.1* was significantly upregulated by fructose, and *RsSTP1.2* and *RsSTP4.2* were significantly downregulated by sucrose.

## Discussion

STP proteins in plants play vital roles in monosaccharide transport and are involved in regulating multiple growth and developmental processes and biotic and abiotic resistance. In recent years, with the availability of various plant genomes, genome-wide and expression analysis of the STP gene family has been reported in many plants, such as *Arabidopsis thaliana* [6], *Oryza sativa* [27, 31], *Solanum lycopersicum* [32], *Pyrus bretschneideri* Rehd) [33], *Brassica oleracea* var. *capitata* L. [34], *Fragaria vesca* [35], *Capsicum annuum* L. [29], *Manihot esculenta* [36], *Triticum aestivum* L. [37] and son on. However, the evolutionary dynamics and functional analysis of the STP gene family in Brassicaceae crops are still largely unknown.

In the present study, a total of 25, 25, 28, 36 and 49 STP-encoding genes were identified from the genomes of *R. sativus*, *B. oleracea*, *B. rapa*, *B. napus* and *B. juncea*, respectively. The same *STP* gene numbers were identified in *R. sativus* and *B. oleracea* even though *R. sativus* (460 Mb and 44,109 coding genes, respectively) [38] and *B. oleracea* (648 Mb and 54,475 coding genes, respectively) [39] have different genome sizes and gene numbers. *B. rapa* (442.9 Mb and 45,985 coding genes, respectively) [40] and *R. sativus* have similar genome sizes and gene numbers, but variant in *STP* gene numbers. Thus, the number of *STP* genes is no direct correlation with genome size and the total number of coding genes in Brassicaceae crops. The same result was also reported by a previous report in Gramineae crops [7]. Tetraploid *B. juncea* (920 Mb and 101,959 coding genes, respectively) [41]*,* which have undergone an allopolyploidization event, as well as *B. napus* (1008 Mb and 100,919 coding genes, respectively) [42], have an almost twice larger genome size and number of coding genes than diploid *R. sativus*, *B. oleracea* and *B. rapa*, and have significantly more *STP* gene numbers were identified*.* This result makes us speculated that a large number of *STP* genes retained after the allopolyploidization event, even though accompanied by gene losses occur in this process.

Segmental and tandem duplication events play a critical role in the expansion and increased functional diversity of the STP gene family. Previous studies indicated that a WGT occurred in the common ancestor of Brassicaceae crops following its divergence from *A. thaliana* [28]*.* In this study, we revealed that most *AtSTP* genes have syntenic pairs in other Brassicaceae species with more than one copy, which is consistent with the polyploidization of these species. Here, segmental duplication was the major force driving the expansion of *STP* genes in Brassicaceae. Additionally, several genes were lost or retained one copy, suggesting that there may have been some variability in the gene loss events during evolution. Previous studies concluded that functionally redundant genes prefer to be lost in the diploidization process occurring after paleopolyploidy events [43]. The tandem duplication *STP10* genes were present in *R. sativus*, *B. rapa, B. oleracea* and two subgenomes of *B. juncea*, indicating that the associated tandem duplication event occurred in the common ancestor.

We revealed physical and chemical properties, diverse gene structures, conserved motifs and phylogenetic analysis of *STP* genes in Brassicaceae crops. The physical and chemical properties of *STP*s showed that there were no significant differences among the six species, which indicated that the STP proteins were conserved among different species. Furthermore, most of the *STP* genes contained four exons and three introns, which is similar to other plant species, such as rice, tomato, and pears [27, 31–33]. A phylogenetic analysis revealed that the STPs in Brassicaceae crops were classified into four groups, consistent with the classification in cassava and Gramineae [7, 36], suggesting that STP proteins are highly conserved across lineages. These results indicated that STP functions are mainly conserved in different species.

Previous studies have produced evidence that *STPs* are involved in plant growth and developmental processes and biotic and abiotic stress responses. The *cis*-acting elements in the promoter regions are always associated with their transcriptional control and function. In the present study, *cis*-acting elements involved in light responsiveness, responding to phytohormones and stresses were also found in the predicted promoter regions of *RsSTPs* (Fig. S4), suggest that these genes might be involved in radish growth and developmental processes and biotic and abiotic stress responses.

In the process of evolution, most of the homologous genes of different species retain the same or similar biological functions, and the expression patterns of genes are often closely related to their functions. In the present study, expression analysis of *RsSTPs* in different tissues showed that *RsSTP4.1* and *RsSTP4.3* were expressed in all tissues and their expression levels were significantly induced under Pb and Cd stress, but not by Cr stress and *A. tumefaciens* infection. *AtSTP4*, which is orthologous in *A. thaliana*, could transport a broad spectrum of monosaccharides [11], and induced by fungal biotroph *Erysiphe cichoracearum* infection [44]. Therefore, *RsSTP4.1* and *RsSTP4.3* might play vital roles in soluble sugar transporters in different tissues of radish and also involved in Pb and Cd stress resistance. However, their biological function needs further experimental verification. *AtSTP4/6/8/9/10/11* were found to be highly expressed in pollen tubes by a previous study [22]. Our present results show that eight genes (*RsSTP2.1/2.2/4.2/6.1/7.2/9.1/9.2/14*) were specifically expressed in flowers, and we conjectured that these genes might be responsible for glucose uptake into pollen tubes. *RsSTP3* and *RsSTP6.2* were highly expressed in seedpods, suggesting that they might be involved in soluble sugar accumulation in this tissue. *STP* genes were reported participate in response to biological stress in other plants. The expression of *BoSTP4b* and *BoSTP12* were up-regulated in cabbage with *Plasmodiophora brassicae* infection [34]. *RsSTP6.2* and *RsSTP13.2* most likely confer radish resistance to *A. tumefaciens* infection by RNA-seq data analysis in the present study. Earlier studies showed that *STP* genes play important roles in various sugar transportation processes [4, 6, 8–19]. We detected that the expression of most genes was not significantly affected by 2% glucose, fructose and sucrose treatment, and five genes were significantly downregulated by at least one treatment (Fig. 9). Only *RsSTP8* and *RsSTP13.1* were upregulated by fructose treatment. Deng et al. also indicated that only *OsST8* was upregulated by fructose treatment in the roots of rice [27]. The transporter of glucose and sucrose in radish roots might be responsible for other sugar transporters.

*RsSTP13.2* was significantly upregulated in resistant plants by *A. tumefaciens* infection but undetectable in susceptible plants and induced by Cd, Cr, Pb, NaCl and PEG-6000 stress, indicating that *RsSTP13.2* is involved in resistance to both biotic and abiotic stress in radish. A previous study also indicated that *STP13* is involved in biotic and abiotic responses and resistance in other plants. In *A. thaliana*, *STP13* maintains low expression under normal conditions, but *STP13* is induced by MYB96 and reabsorbs the monosaccharides that are released by damaged cells under saline conditions [45, 46]. The upregulation of *AtSTP13* deprivation of extracellular sugar levels, which is used as an energy source for pathogens, enhances antibacterial defence [47]. In addition, *AtSTP13* is also involved in resistance to *Botrytis cinerea* by affecting glucose transport [23]. In *Phaseolus vulgaris* L., both excess zinc and iron/zinc deficient stress significantly upregulate the expression of *STP13* in the shoot, while excess zinc stress induced and iron/zinc deficient stress decreased its expression in roots [26].

## Conclusions

The present study provides insights into the evolutionary patterns of the STP gene family in Brassicaceae genomes and provides a theoretical basis for future functional analysis of *STP* genes in Brassicaceae crops. *RsSTP13.2* may serve as a candidate gene to improve the biotic and abiotic resistance of plants through transgenic technology.

## Methods

### Data sources and identification of *RsSTP* family genes

The *R. sativus* ‘Xin-li-mei’ whole-genome sequence [38] was used to identify the *RsSTP* family genes. The whole-genome sequences of *B. napus* [42]*, B. juncea* [41], *B. rapa* [40], *B. oleracea* [39] and *A. thaliana* were obtained from the BNPIR database (http://cbi.hzau.edu.cn/cgi-bin/rape/download_ext), NCBI database (https://www.ncbi.nlm.nih.gov/bioproject/PRJNA550308), BRAD database (http://brassicadb.cn/#/), Ensembl (http://plants.ensembl.org/info/data/ftp/index.html) and TAIR database (http://www.arabidopsis.org/), respectively. Hmmsearch integrated in HMMER software [48] was used to identify *STP* gene family members with the hidden Markov model (HMM) profile of Sugar_tr (PF00083) obtained from the Pfam database (http://pfam.xfam.org). Then, the SMART database (http://smart.embl-heidelberg.de) and Batch CD-Search (https://www.ncbi.nlm.nih.gov/Structure/bwrpsb/bwrpsb.cgi) were further used to confirm the genes that were obtained with highly conserved sugar_tr domains and MFS_STP domains. The *STP* genes of *B. napus, B. juncea, B. rapa, B. oleracea* and *R. sativus* were named according to their phylogenetic relationship with *A. thaliana STP* genes*.*

### The phylogenetic tree construction

For phylogenetic tree construction, first, all the STP full-length protein sequences of six Brassicaceae species were aligned by using the MUSCLE program [49]. Then, MEGAX [50] was used to construct a neighbour-joining tree using the Jones-Taylor-Tornton (JTT) model with 1000 bootstrap replicates. Additionally, uniform rates and homogeneous lineages were adopted, and partial deletion with a site coverage threshold of 70% was given for gaps/missing data.

### Sequence properties, conserved motifs and gene structure analyses

The Biopython module Bio.SeqUtils. ProtParam of Python language was used to calculate the molecular weight, aromaticity and other physical and chemical properties of STP proteins. The R script was used to compare the differences among different species using ggpubr with ‘anova’ methods. The MEME suite [51] was used to identify the conserved motifs of STP proteins with the following parameters: the maximum number to be found was set to 15, and the motif length was set to 8–100 bp. The Pfam domains of motifs were identified in the Pfam database (http://pfam.xfam.org). The gene structures information containing extron and intron position were obtained from GFF file using python script. The TBtools program [52] was used to visualize gene structures and conserved motifs.

### Tandem duplications and syntenic analysis of *STP* genes

Tandem genes in *A. thaliana* and other species were defined as those genes that were separated by ten or fewer genes. The Multiple Collinearity Scan toolkit (MCScanX) [53] was used to identify syntenic duplication events between *A. thaliana* and other species, with the default parameters. The synteny relationship of *STP* genes was visualized using TBtools software [52]. The subgenomes of *B. napus* and *B. juncea* were calculated separately.

### *Cis*-acting element analyses of *RsSTPs* and its transcriptional profiles in RNA-seq data

The *cis*-acting elements in the potential promoter region (the upstream 1.5 kb sequence starting from the start codon) of the radish *STP* genes were identified using the PlantCARE program (http://bioinformatics.psb.ugent.be/webtools/plantcare/html/).

The *RsSTP* expression profiles of different tissues (root, leaf, bolting, flower, silique and callus), their response to heavy metals (cadmium, chromium and lead) and *Agrobacterium tumefaciens* infection were analysed based on public transcriptome data [30, 54–57]. The fragments per kilobase of transcript per million mapped reads (FPKM) data were log_2_ transformed, and a heatmap was created using TBtools [52].

### Plant materials and stress treatments

Seeds of radish cultivar ‘Xin-li-mei’ purchased from Jingyan Yinong (Beijing) Seed Sci-Tech Co., Ltd. were surface-sterilized in 1% NaClO and incubated at 22 °C for 2 d in darkness. The germinated seeds were sown into plastic pots and incubated in a growth chamber at a 16 h day (22 °C)/8 h night (20 °C) cycle. Seedlings were watered as needed with half-strength Hoagland’s nutrient solution. Plants at the three true leaf stages were used for subsequent abiotic stress and sugar treatment. For the simulated salinity and drought stress treatments, the seedlings were subjected to 1.5% NaCl and 20% PEG-6000 for 3 h, respectively. For sugar treatments, the seedlings were subjected to 2.0% glucose, sucrose and fructose solution for 3 h. The seedlings treated with sterile water were used as a control. Eight plants were used as biological replicates, and the roots were collected. The samples were frozen in liquid nitrogen immediately after collection and stored at -80 °C for RNA extraction. The collection of plant material, is in compliance with relevant institutional, national, and international guidelines and legislation.

### RNA extraction, reverse transcription quantitative polymerase chain reaction (RT-qPCR) analysis of *RsSTPs*

Total RNA was extracted from different radish samples with a Quick RNA Isolation Kit (Huayueyang, Beijing, China) according to the manufacturer's instructions. A total of 800 ng of high-quality total RNA was used to synthesize first-strand cDNA with PrimeScript® Reverse Transcriptase (Takara Biotechnology, Dalian, China). The SYBR Green qPCR kit (Takara Biotechnology) was used for RT-qPCR in a Stratagene Mx3000P thermocycler (Agilent, Santa Clara, CA, USA). *RsGAPDH* was utilized as an internal control, and the relative expression levels of *RsSTPs* were calculated with the 2^−ΔΔCt^ method [58, 59]. The primer used for RT-qPCR is shown in Table S1.

## Supplementary Information

**Additional file 1: Figure S1.** Distribution of STP genes on chromosomes. The line on the green bars indicates the location of STP genes on chromosomes. The left values corresponding to the scales indicate physical chromosomes. The red genes indicated tandem duplicated genes.

**Additional file 2: Figure S2.** The percent of exons numbers of STP genes.

**Additional file 3: Figure S3.** The percentage of segmental or tandem duplication genes in five Brassicaceae crops.

**Additional file 4: Figure S4.** The types and numbers of cis-acting elements identified in the promoters of *RsSTP* genes.

**Additional file 5: Table S1.** The identified STP genes in six Brassicaceae species. **Table S2.** The physical and chemical properties of STP proteins.

**Additional file 6: Table S3.** Primers used for RT-qPCR in the present study.

## Data Availability

The *R. sativus* ‘Xin-li-mei’ whole-genome sequence was downloaded from CNCB database with accession number of GWHANWD00000000 (https://ngdc.cncb.ac.cn/gwh/Assembly/9797/show). The genome and its annotation file of *B. napus* version ZS11 were obtained from BnPIR database (http://cbi.hzau.edu.cn/cgi-bin/rape/download_ext). The genome and its annotation file of *B. juncea* were obtained from NCBI with the accession PRJNA550308 (https://www.ncbi.nlm.nih.gov/bioproject/PRJNA550308). The *B. rapa* genome version 3.5 information was downloaded from BRAD (http://brassicadb.cn/#/Download/). The *B. oleracea* genome was downloaded from Ensembl (http://plants.ensembl.org/Brassica_oleracea/Info/Index). The *A. thaliana* genome was obtained from TAIR database (http://www.arabidopsis.org/). The RNA-seq reads in different tissues (root, leaf, bolting, flower, silique and callus) of *R. sativus* are available at NCBI Sequence Read Archive (SRA, http://www.ncbi.nlm.nih.gov/Traces/sra) (PRJNA413464). The RNA-seq raw data of control and heavy metal stress treatments were downloaded from SRA with accession numbers: SRX256970 (CK), SRX862647 (Cr600), SRX263753 (Pb1000) and SRX824523 (Cd200). The RNA-seq raw data of control and *Agrobacterium tumefaciens* treatments were downloaded from SRA with accession numbers: SRX9205566 (control 18 line), SRX9205567 (tumor 18 line), SRX9205568 (control 19 line) and SRX9205569 (tumor 19 line).

## Abbreviations

STPs: Sugar Transporter Proteins
SWEETs: Sugars Will Eventually Be Exported Transporters
MSTs: Monosaccharide Transporters
SUTs: Sucrose Transporters
VGT: Vacuolar Glucose Transporter
TMT: Tonoplast Monosaccharide Transporter
GlcT: Plastidic Glucose Transporter
SGB1: Suppressor of G Protein Beta 1
PLT: Polyol Transporter
INT: Inositol Transporter
ERD6L: Early Response to Dehydration-6-Like
Cd: Cadmium
Cr: Chromium
Pb: Lead
JTT: Jones-Taylor-Tornton
MCScanX: Multiple Collinearity Scan toolkit
RT-qPCR: Reverse Transcription Quantitative Polymerase Chain Reaction
