# Supplementary material for: Genome-wide identification of sugar transporter gene family in Brassicaceae crops and an expression analysis in the radish
Source: BMC Plant Biol. 2022 May 18;22:245. doi: 10.1186/s12870-022-03629-2 (PMC9115943; doi:10.1186/s12870-022-03629-2)
Supplement: Supplementary file 1 — Additional file 1: Figure S1. Distribution of STP genes on chromosomes. The line on the green bars indicates the location of STP genes on chromosomes. The left values corresponding to the scales indicate physical chromosomes. The red genes indicated tandem duplicated genes. [file 12870_2022_3629_MOESM1_ESM.pdf]

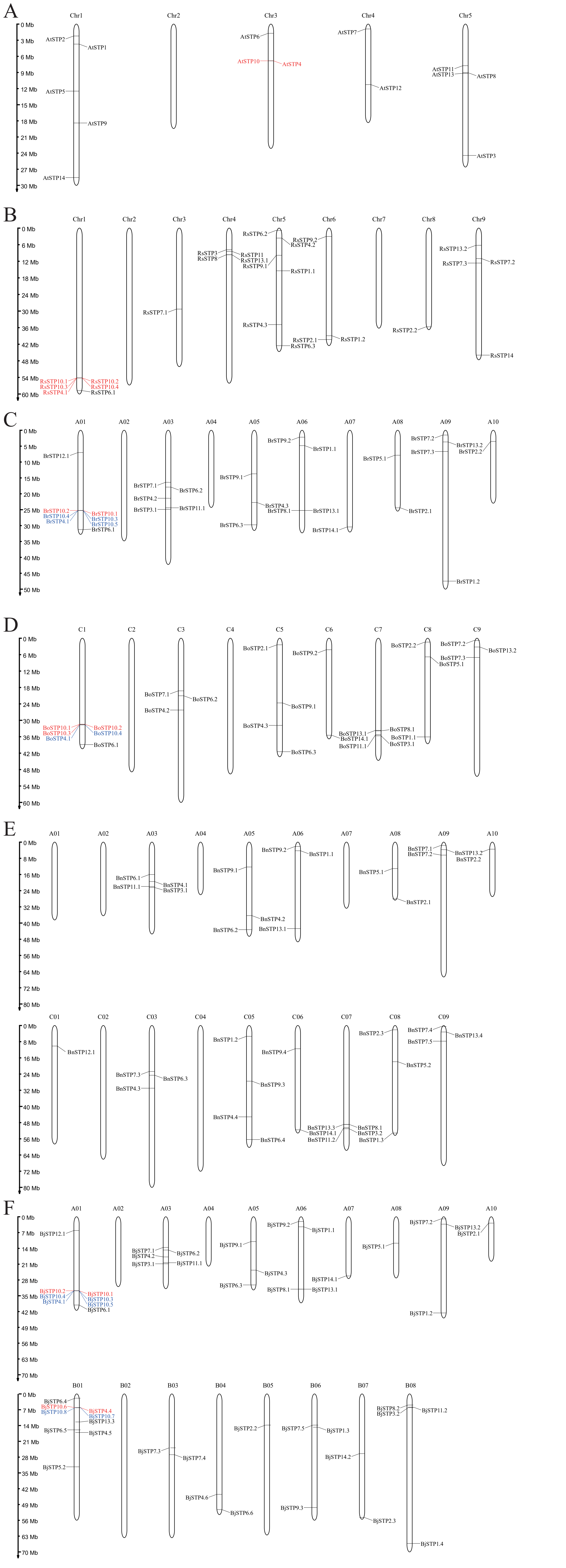

Figure S1. Distribution of STP genes on chromosomes. The line on the green bars indicates the location of STP genes on chromosomes. The left values corresponding to the scales indicate physical chromosomes. The red genes indicated tandem duplicated genes.
