# Supplementary figures and images for: Genome-wide identification of sugar transporter gene family in Brassicaceae crops and an expression analysis in the radish
Source: BMC Plant Biol. 2022 May 18;22:245. doi: 10.1186/s12870-022-03629-2 (PMC9115943; doi:10.1186/s12870-022-03629-2)

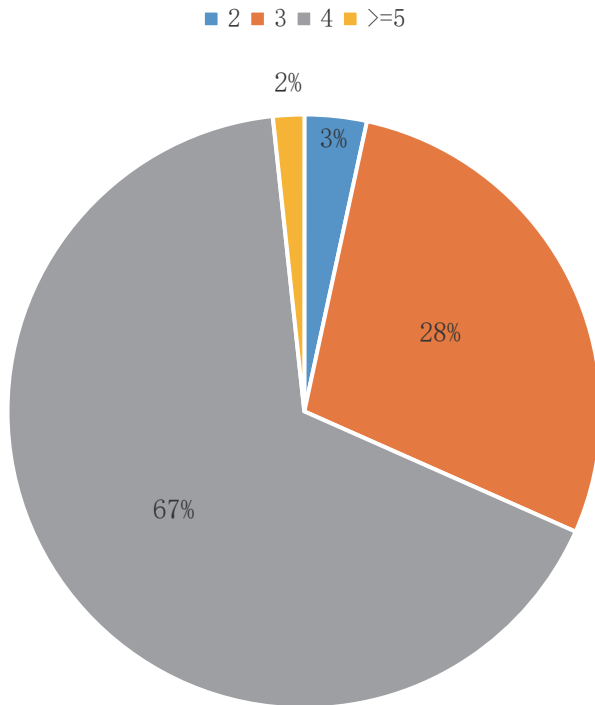

Figure S2. The percent of exons numbers of STP genes.

Supplement: Supplementary file 2 — Additional file 2: Figure S2. The percent of exons numbers of STP genes. [file 12870_2022_3629_MOESM2_ESM.pdf]

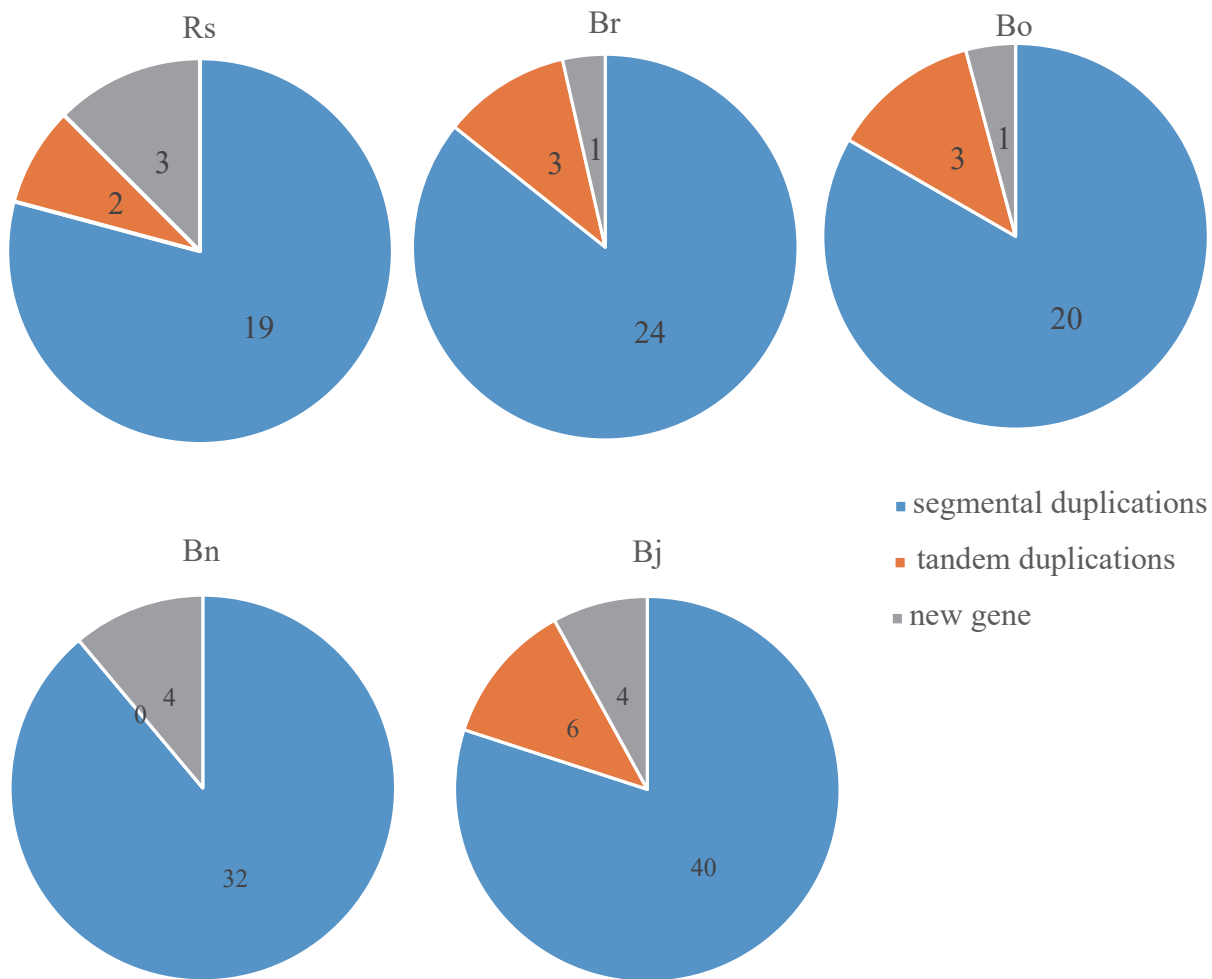

Figure S3. The percentage of segmental or tandem duplication genes in five Brassicaceae crops.

Supplement: Supplementary file 3 — Additional file 3: Figure S3. The percentage of segmental or tandem duplication genes in five Brassicaceae crops. [file 12870_2022_3629_MOESM3_ESM.pdf]

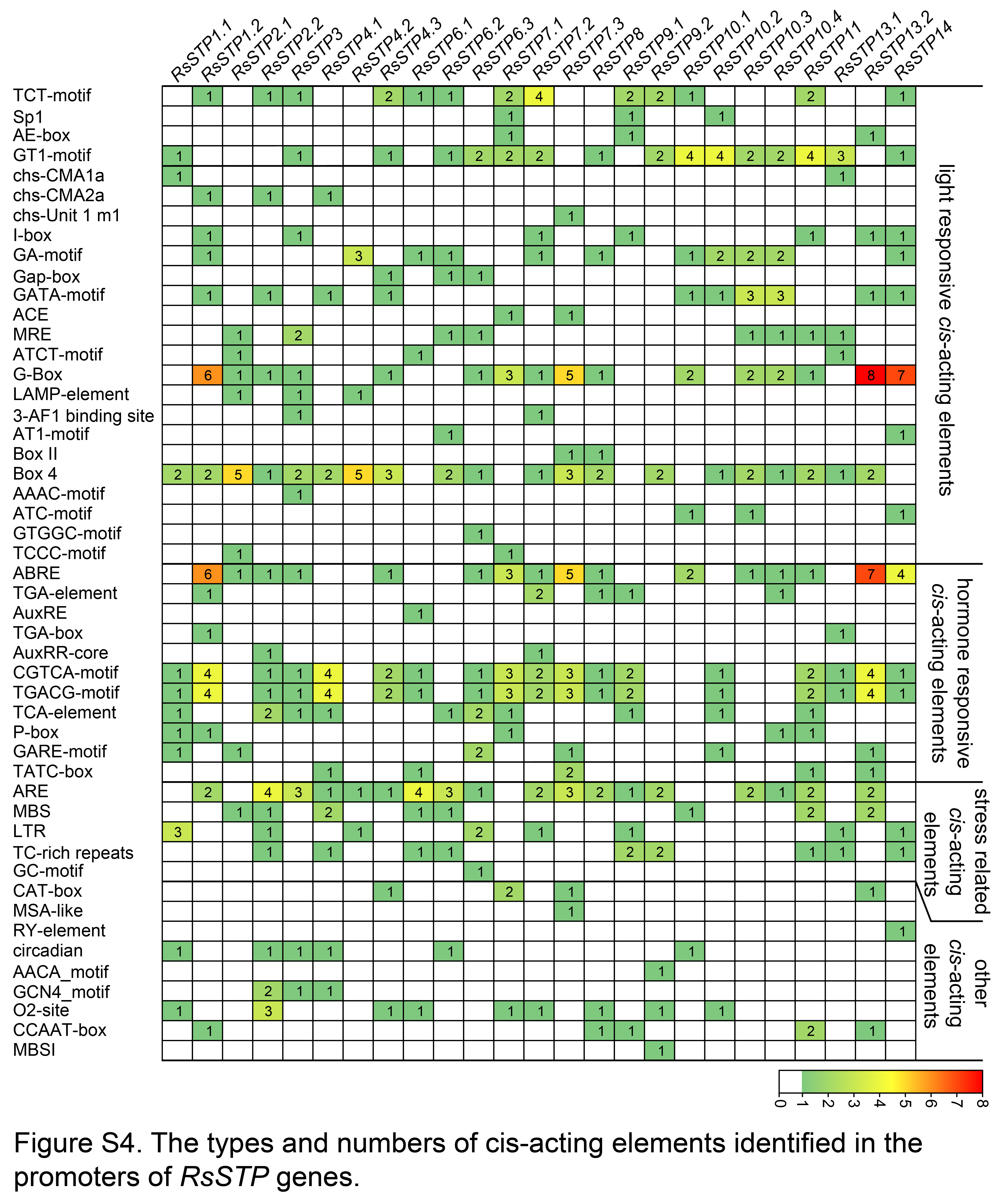

Supplement: Supplementary file 4 — Additional file 4: Figure S4. The types and numbers of cis-acting elements identified in the promoters of RsSTP genes. [file 12870_2022_3629_MOESM4_ESM.tif]
